# Starling-Behavior-Inspired Flocking Control of Fixed-Wing Unmanned Aerial Vehicle Swarm in Complex Environments with Dynamic Obstacles

**DOI:** 10.3390/biomimetics7040214

**Published:** 2022-11-26

**Authors:** Weihuan Wu, Xiangyin Zhang, Yang Miao

**Affiliations:** 1 Faculty of Information Technology, Beijing University of Technology, Beijing 100124, China; 2 Engineering Research Center of Digital Community, Ministry of Education, Beijing 100124, China; 3 Faculty of Materials and Manufacturing, Beijing University of Technology, Beijing 100124, China

**Keywords:** starlings, collective motion, fixed-wing UAV swarm, local following, obstacle avoidance

## Abstract

For the sake of accomplishing the rapidity, safety and consistency of obstacle avoidance for a large-scale unmanned aerial vehicle (UAV) swarm in a dynamic and unknown 3D environment, this paper proposes a flocking control algorithm that mimics the behavior of starlings. By analyzing the orderly and rapid obstacle avoidance behavior of a starling flock, a motion model inspired by a flock of starlings is built, which contains three kinds of motion patterns, including the collective pattern, evasion pattern and local-following pattern. Then, the behavior patterns of the flock of starlings are mapped on a fixed-wing UAV swarm to improve the ability of obstacle avoidance. The key contribution of this paper is collective and collision-free motion planning for UAV swarms in unknown 3D environments with dynamic obstacles. Numerous simulations are conducted in different scenarios and the results demonstrate that the proposed algorithm improves the speed, order and safety of the UAV swarm when avoiding obstacles.

## 1. Introduction

Group formation is widespread, such as in schools of fish, flocks of birds and swarms of honeybees when they perform seasonal migration, travel to food sources or avoid predators [1,2,3]. A paradigmatic case of flocks of birds is provided by starlings [4,5], which are a highly gregarious species. Grouping has several benefits: it reduces the chance of an individual being attacked and decreases the risk of being exposed to hazards. Collective motions in flocks have intrigued scientists in various research fields, including control engineering and computer science. The study of collective motion began in the 1980s with the development of computer simulations. In 1987, Reynolds proposed a classical collective model [6] (called the Boid model), which is consisted of three rules: (1) Separation: each agent avoids colliding with nearby neighbors; (2) Cohesion: each agent stays close to nearby neighbors; (3) Alignment: each agent matches velocity with nearby neighbors. In 1995, Vicsek proposed a collective model [7] (called the Vicsek model) with self-propelled particles driven by constant velocity. The direction of particles is obtained by calculating the average direction of their neighbors. The Vicsek model accomplishes the self-organization and velocity consensus of multi-agent systems. In 2002, Couzin proposed a collective model [8] in three-dimensional space that divides the perceptual area of agents into three different parts. These three parts are the exclusion area, the alignment area and the attractive area, which correspond to the separation, cohesion and alignment rules. In 2006, Olfati-Saber presented a flocking algorithm [9] for free-flocking and constrained flocking. Each agent in constrained flocking has a control input consisting of a formation term, an obstacle avoidance term and a lead-following term. The flocking algorithm results in self-organized flocking behavior. In 2007, Tanner proposed a flocking algorithm [10] in fixed and switching networks. The network of multi-agent systems remains constantly connected regardless of switching topology. In the last decade, researchers discovered that most collective behavior of animals has the following advantages: decentralization, following the sample rules, flexibility. Therefore, a great deal of research is also inspired by the collective behavior of animals. Duan et al. [11] presented a novel swarm intelligence optimizer based on the collective behavior of pigeons, after which authors applied this pigeon-inspired optimization for solving air robot path planning problems. Zhang et al. [12] proposed an enhanced fruit fly optimization algorithm based on quantum theory and the collective behavior of fruit flies. In addition, the proposed algorithm was also adopted to unmanned aerial vehicle path planning problems in the three-dimensional environment. Zhou et al. [13] explored the problem of bionic flight control in an aircraft formation. By analyzing the formation flight mechanism of wild geese during migration, this research work designed a tight configuration for formation and a control method for multiple aircrafts. Xie et al. [14] proposed a starling swarm coordination algorithm inspired by the collective behavior of starlings. The algorithm adopted the thought of decentralization and self-organization to realize the behavior evolution process of collective motion from disorder to order.

In recent years, the unmanned aerial vehicle (UAV) has become widely used in military and civilian fields such as agriculture by virtue of the development of autonomy [15,16,17]. Compared with a single UAV, multiple UAVs are able to execute missions such as surveillance and reconnaissance simultaneously, which greatly improves execution efficiency [18]. Obstacles are one of the biggest threats to flight for multi-agent systems when executing missions. Most existing methods of obstacle avoidance are divided into known environment and unknown environment according to whether there is prior information. The typical motion planning method in a known environment includes the A* search algorithm [19], particle swarm optimization (PSO) [20,21] algorithm and model prediction control (MPC) method [22]. The reactive method for obstacle avoidance includes the artificial potential field (APF) method [23,24]. These methods are intuitive and convenient to implement, but the generated path may fall into local minima in some cases. Additionally, a deep-reinforcement-learning-based reactive online decision-making mechanism is applied in [25] to figure out the problem of obstacle avoidance, but this research only involves a single UAV. Some research [26,27,28] only considers a sparse obstacle environment or only stays in a two-dimensional environment, which is a disparate scenario compared with the environment in which a UAV swarm executes missions in reality. Unlike an insect swarm or a terrestrial animal (e.g., horse or wolf) swarm that consist of hundreds of individuals, a flock of starlings always consists of thousands of members, which makes starling flocks more valuable to explore. The collective motion of starlings emerges as the result of the local interaction between nearby neighbors [29], instead of relying on centralized coordination. Additionally, a flock of starlings is able to evade threats in an orderly fashion [30]. In groups, potential threats may be spotted earlier and information may be spread faster than among solitary individuals [31]. Enlightened by the collective evasion of starlings [30,32], a turning control is adopted to avoid obstacles in this paper. The UAV swarm turning control denotes that after a UAV in the group has turned, other UAVs reach a consensus state of turning to avoid threats through their perception and judgment even though they have not detected the threat. Therefore, fast information transmission in the UAV swarm is vital for obstacle avoidance.

In this paper, a control framework is proposed to solve the obstacle avoidance problem of a UAV swarm in an unknown environment with dynamic threats. This distributed control framework mimics behavioral patterns of the flocking of starlings when they evade threats. In order to improve the performance of the UAV swarm, three major patterns of flocks of starlings have been applied to the UAV swarm. Based on these patterns, the UAV swarm can aggregate and reach a consistent state immediately in the environment without obstacles; when the UAV swarm encounters obstacles, the turning curvature of the path and the consistency of the UAV swarm can be satisfied simultaneously.

The rest of paper is organized as follows. Section 2 discusses the behavior mechanism of starlings and presents the behavioral patterns of the flock of starlings in detail. Section 3 defines the model of the UAV and mapping behavioral patterns on the UAV swarm. In Section 4, simulations and comparisons are depicted to demonstrate the effectiveness of the proposed algorithm. Conclusions are drawn in Section 5.

## 2. Model of Starling Behavior

### 2.1. The Behavioral Mechanism of Starlings

**Figure 1:**
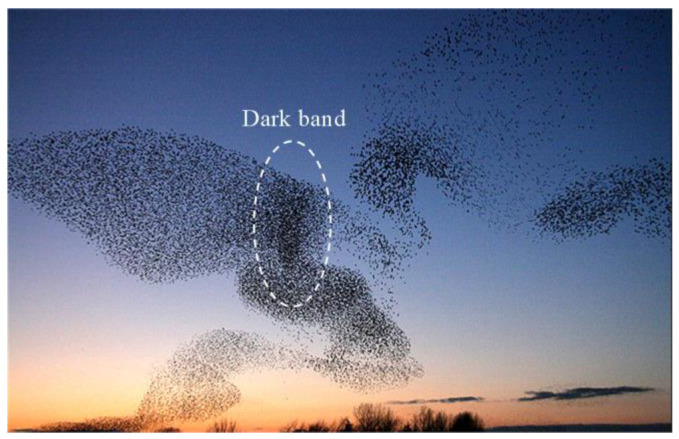
A flock of starlings in the sky [35] copyright: 5434760037420.

**Figure 2:**
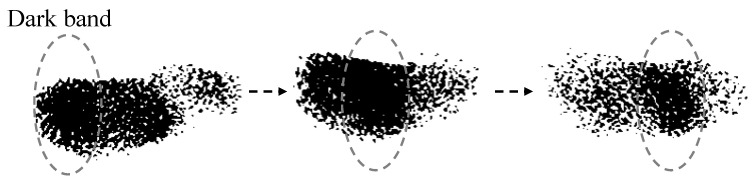
Schemes follow the same formatting [5] copyright: © 2015, The Author(s).

When a flock of starlings confronts threats, individuals near the threat become alert and take action. Because the flock of starlings is too large, it is inevitable that individuals far from the threat will not be aware of danger in the first place. Therefore, fast transmission of information in groups can increase the survival rate of the whole flock. Those individuals that detect threats earlier are selected as local leaders of their neighbor unit to transmit information [33]. The information transmission appears as a wave of agitation in the starling flock [34]. As shown in Figure 1 and Figure 2, the movement of a dark band called an agitation wave generates from the position of the threat and propagates away from it. Biological research about collective behavior indicates individuals on the far side of group will be alerted earlier than if they were alone.

Flocks of starlings have the capability to gather immediately through local information interaction. In the starling flock, the orientation of each starling is not only decided by their self-information but also decided by their neighbors [29]. Each starling reaches the velocity consensus with its neighbors, so that the entire flock will form a uniform movement.

**Figure 3:**
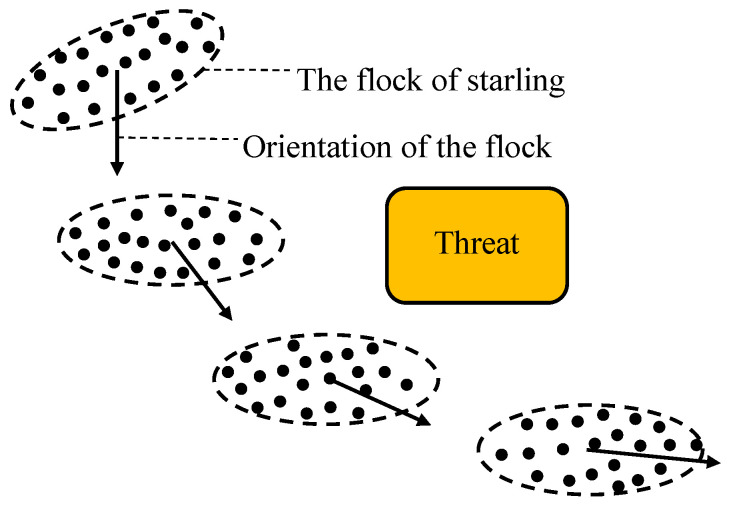
Schematic of the turning flock on the horizontal plane.

As shown in Figure 3, a flock of starlings will change orientation to avoid collision with threats. The geometric shape of the flock on the plane remains approximately constant during turns.

### 2.2. Bevioral Patterns Based on Bevioral Mechanisms of Starlings

Based on analyses in Section 2.1, there are three major patterns in flocks of starlings [36]: the collective pattern, evasion pattern and local-following pattern. In obstacle-free areas, the tendency of each starling to imitate its neighbors produces a global collective state, i.e., the flock of starlings in the collective pattern. In the obstacle environment, starlings that have detected obstacles will switch to the evasion pattern to avoid obstacles, while those starlings that are not aware of threats will follow the starlings that have switched to the evasion pattern [36]. Fast information transmission allows coordination of motion and effective response to the complex environment.

#### 2.2.1. Collective Pattern

**Figure 4:**
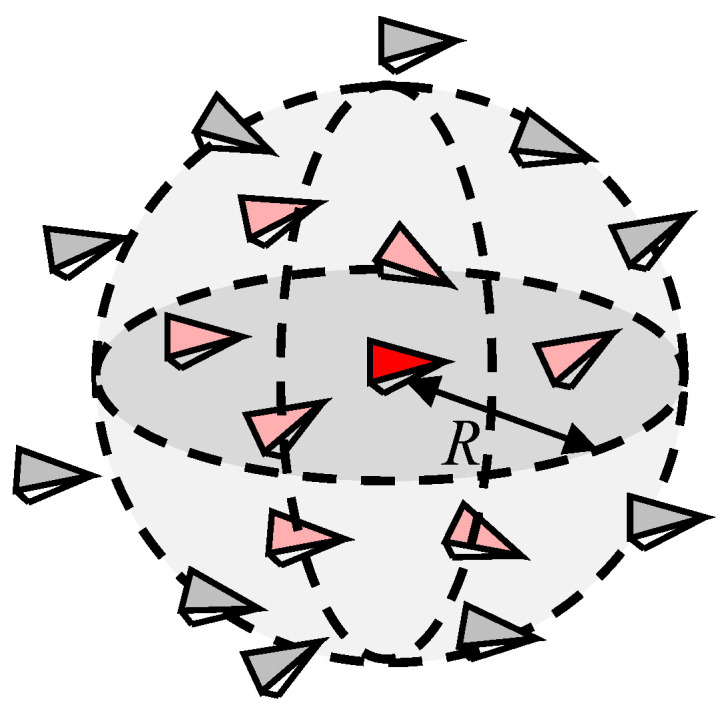
Schematic of the neighbor set, the red marker represents the individual *i* and pink markers represent neighbors of individual *i*.

In reality, constrained by a limited sensing ability, starlings can only communicate with neighbors in a fixed distance (as shown in Figure 4, the red marker represents the individual *i* and pink markers represent neighbors of individual *i*). The neighbor set of the individual *i* is denoted by

(1) $$N_{i}(t)=\{j:\left\|x_{i}-x_{j}\right\|\leq R,j=1,2,\ldots ,N,j\neq i\}$$

where $x_{i},x_{j}\in R^{3}$ are the position of individual *i* and *j*, respectively. ‖•‖ represents the Euclidean distance and *R* is the sensing radius of each individual.

According to research and data about biological collective behavior, an individual in a flock of starlings also follows cohesion, alignment and separation rules. Each starling shares velocity and location information with its neighbors to decide the actions that need to be performed at that moment, which can be described as follows:

(2) $$f_{i}^{col}=f_{i}^{pos}+f_{i}^{vel}$$

where the neighbor interaction term $f_{i}^{col}\in R^{3}$ consists of the position coordination term $f_{i}^{pos}\in R^{3}$ and the velocity coordination term $f_{i}^{vel}\in R^{3}$.

If a flock of starlings gathers too closely, individuals will repel each other to avoid collision. However, starlings will also gather more closely when the distance between two neighbors is too large. Thus, the position coordination term is described as follows:

(3) $$f_{i}^{pos}=\sum_{j\in N_{i}(t)}\left(\frac{1}{\left\|x_{i}-x_{j}\right\|}-\frac{1}{{\left\|x_{i}-x_{j}\right\|}^{3}}\right)$$

The alignment rule states that the velocity of a starling should keep consistent with its neighbors to guarantee the order of the flocking system. Thus, the velocity alignment term is defined as follows:

(4) $$f_{i}^{vel}=-\sum_{j\in N_{i}(t)}\left(v_{i}-v_{j}\right)$$

where $v_{i},v_{j}\in R^{3}$ are the velocity of individual *i* and *j*, respectively.

#### 2.2.2. Evasion Pattern

**Figure 5:**
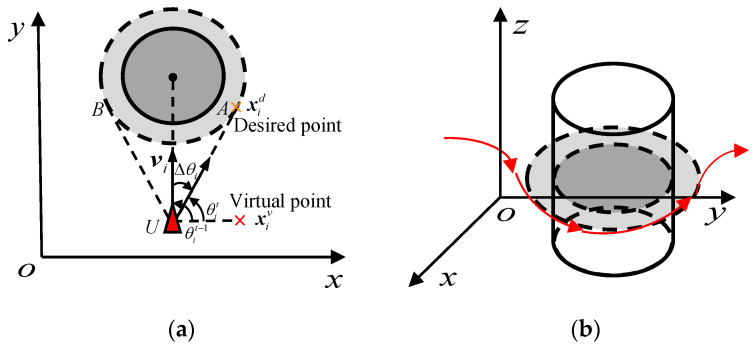
Diagram of the threat. (**a**) is in *x*-*y* plane, while (**b**) is in the 3D space, the dark grey circled area represents the real obstacle while the radius of the light grey circle area is the expected distance from individual *i* to the center of obstacle. The red arrows in (**b**) represent the motion direction of the individual *i*.

When a flock of starlings encounters obstacles, individuals avoid colliding with obstacles by changing their orientation, which is called the tangential navigation schema [37]. As shown in Figure 5, the dark grey circled area represents the real obstacle while the radius of the light grey circle area is the expected distance from individual *i* to the center of obstacle. The red arrows in Figure 5b represent the motion direction of the individual *i*. The velocity vector $v_{i}$ of the individual *i* is in the area UAB, which indicates that if the individual *i* keeps the original heading angle $\theta_{i}^{t-1}$, a collision with the obstacle will happen.

Consequently, the individual *i* will align its heading angle to the tangent direction of the virtual scope of the obstacle. Predefine a parameter to describe the direction of rotation as follows:

(5) $$c_{r}=\left\{ \begin{array}{l} -1,\;\mathrm{for}\;\mathrm{clockwise}\;\mathrm{rotation}\\ 1,\;\mathrm{for}\;\mathrm{counter}-\mathrm{clockwise}\;\mathrm{rotation}\; \end{array}\right.$$

Supposing that the turning angle of individual *i* is $\Delta \theta_{i}$, the new heading angle pointed at the desired point can be calculated as follows:

(6) $$\theta_{i}^{t}=\theta_{i}^{t-1}+c_{r}\cdot \Delta \theta_{i}$$

The position of virtual point $x_{i}^{v}\in R^{3}$ is a projection of the position of the desired point $x_{i}^{d}\in R^{3}$ on the *x*-axis. So, $x_{i}^{d}$ is calculated as follows:

(7) $$x_{i}^{d}=x_{i}+\left[\begin{array}{ccc} \cos\theta_{i}^{t} & -\sin\theta_{i}^{t} & 0\\ \sin\theta_{i}^{t} & \cos\theta_{i}^{t} & 0\\ 0 & 0 & 1 \end{array}\right](x_{i}^{v}-x_{i})$$

Therefore, the pattern of evasion is donated as follows:

(8) $$f_{i}^{eva}=-\frac{x_{i}-x_{i}^{d}}{\left\|x_{i}-x_{i}^{d}\right\|}$$

#### 2.2.3. Local-Following Pattern

**Figure 6:**
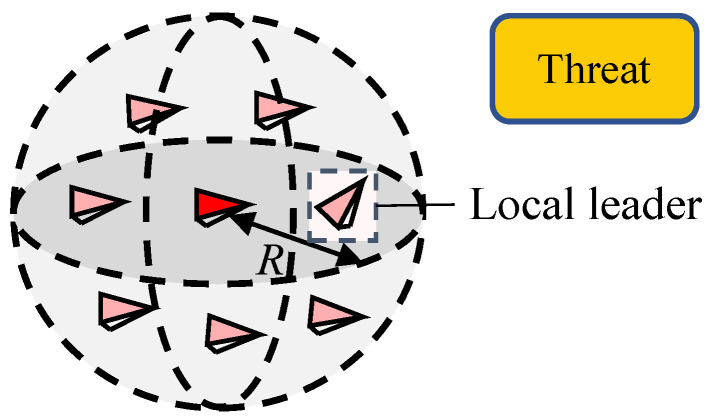
Schematic of the selection of local leader, the red marker represents the individual *i* and pink markers represent neighbors of individual *i*.

In the free motion of the flocking system, the information flow is mainly conducted by averaging states of all the nearby neighbors. However, when an emergency occurs, a more efficient method to reduce the risk is to follow a single local leader within the neighbor set [38,39]. The single local leader is the individual that has detected threats and has already taken action (as shown in Figure 6).

In order to select an individual as the local leader, the influence of neighbor *j* on individual *i* (the evaluation index) needs to be calculated as follows:

(9) $$C_{ij}=c_{1}\frac{1}{\left\|x_{i}-x_{j}\right\|}\cdot c_{2}(\frac{v_{i}}{\left\|v_{i}\right\|}-\frac{v_{j}}{\left\|v_{j}\right\|})$$

where $c_{1}$ and $c_{2}$ are the coefficients of the influence of position and velocity, respectively. According to the value of the evaluation index, select the individual $l_{i}$ with the most drastic change in motion state in the neighbor set $N_{i}(t)$ as follows:

(10) $$l_{i}=\{\max C_{ij},C_{ij}\geq C^{*},j\in N_{i}(t)\}$$

In addition, a threshold value of $C^{*}$ is designed to decide whether to switch to the local-following pattern:

(11) $$C^{*}=e^{-\alpha \varphi_{i}}$$

where $\varphi_{i}$ is the order parameter of individual *i* and its neighbors. The coefficient α is able to adjust the sensitivity of the group to the order parameter. Indeed, the larger the value of α is, the more sensitive of the flock is, which indicates that the flock is more likely to adopt the local-following pattern. The definition of $\varphi_{i}$ is as follows:

(12) $$\varphi_{i}=\frac{1}{N_{i}+1}\left\|\sum_{j=1}^{N_{i}}\frac{v_{j}}{\left\|v_{j}\right\|}\right\|$$

where $N_{i}$ is the number of individuals in the neighbor set $N_{i}(t)$.

Thus, the pattern of local-following is presented as follows:

(13) $$f_{i}^{lof}=-(v_{i}-v_{l_{i}})$$

## 3. Starling-Behavior-Inspired Flocking Control for UAV Swarm

In this section, the model of the fixed-wing UAV is set up first. The rest of section describes the interaction among fixed-wing UAVs and the obstacle avoidance process that mimic flocks of starlings in detail. In order to simplify the setting of the model, suppose that each UAV is equipped with a GPS, a wireless communication device and a sensor measuring distance (assume that the measurements are without noise), so that each UAV is able to locate and communicate with other UAVs within the sensing radius, as well as detect obstacles.

### 3.1. Model of UAV

Consider a multi-UAV system with *n* UAVs. Let $x_{i},y_{i},h_{i},\psi_{i},V_{i}$ denote the *x* position, *y* position, altitude, heading angle and forward velocity of *i*-th (*i* = 1, 2,…, *n*) UAV in the global coordinate system, respectively. The basic kinematic model of a fixed-wing UAV in a three-dimensional space is described as follows:

(14) $$\begin{array}{l} {\dot{x}}_{i}=V_{i}\cos\psi_{i}\\ {\dot{y}}_{i}=V_{i}\sin\psi_{i}\\ {\dot{V}}_{i}=\frac{1}{\tau_{v}}(V_{i}^{c}-V_{i})\\ {\dot{\psi }}_{i}=\frac{1}{\tau_{\psi }}(\psi_{i}^{c}-\psi_{i})\\ {\ddot{h}}_{i}=-\frac{1}{\tau_{\dot{h}}}{\dot{h}}_{i}+\frac{1}{\tau_{h}}(h_{i}^{c}-h_{i}) \end{array}$$

where $V_{i}^{c},\psi_{i}^{c},h_{i}^{c}$ are the command inputs of velocity, heading angle and altitude to the corresponding autopilots, respectively. $\tau_{v},\tau_{\psi },(\tau_{\dot{h}},\tau_{h})$ are positive time constants for the velocity, heading angle and altitude response with respect to the corresponding command inputs, respectively.

In a practical setting, a real fixed-wing UAV’s $V_{i},{\dot{\psi }}_{i},{\dot{h}}_{i}$ should satisfy the following constraints:

(15) $$\begin{array}{l} v_{\min}\leq V_{i}\leq v_{\max}\\ \left|{\dot{\psi }}_{i}\right|\leq \frac{n_{\max}g}{V_{i}}\\ \lambda_{\mathrm{glide}}\leq {\dot{h}}_{i}\leq \lambda_{\mathrm{climb}} \end{array}$$

where $v_{\min},v_{\max}$ are the minimum and maximum forward velocity; $n_{\max}$ is the maximum lateral overload; $\lambda_{\mathrm{climb}}>0$ is the maximum climbing velocity; $\lambda_{\mathrm{glide}}<0$ is the minimum gliding velocity; *g* = 10 m/s^2^ is the gravitational acceleration. Due to constrains of velocity, the fixed-wing UAV cannot hover or fly backwards.

### 3.2. Collision Prediction Mechanism

In a multi-obstacle environment, there may exist an obstacle that will not be collided with if the UAVs do not change their orientation, which implies that the UAVs need not take action to avoid this obstacle. Therefore, a collision prediction mechanism is introduced to predict whether a collision with an obstacle will occur. Based on assumptions made at the begin of this section, a UAV *i* is able to sense the distance between itself and an obstacle as follows:

(16) $$R_{i}^{obs}=\left\|q_{i}-q_{m}^{obs}\right\|$$

where $q_{m}^{obs}\in R^{3}$ is the position of *m*-th obstacle (*m* = 1, 2,…, *k*, *k* is the number of obstacles).

**Figure 7:**
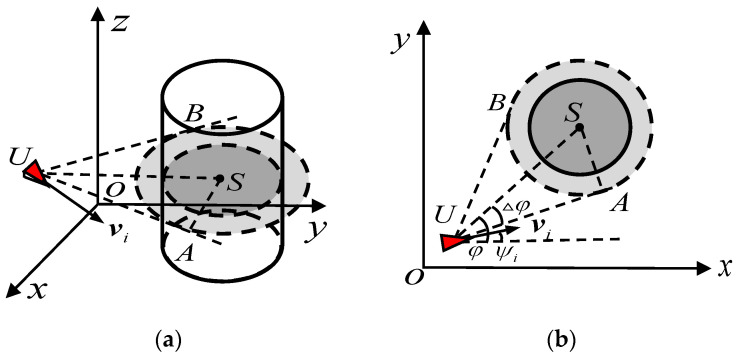
The collision prediction between the UAV and obstacle. (**a**) is in the 3D space, while (**b**) is in the *x*-*y* plane, the red marker represents the individual *I*, the virtual points A and B are the edge points of scope of the obstacle, $\bar{UA},\;\bar{US}$ and $\bar{SA}$ represent the length of line segment UA, US and SA, respectively.

When the desired distance $R_{d}$ between the UAV and obstacle is less than $R_{i}^{obs}$, the collision prediction will start. As shown in Figure 7, the virtual points A and B are the edge points of scope of the obstacle. Supposing that the radius of the virtual zone of the obstacle is $r_{obs}$, the mathematical description of the obstacle effect region is as follows:

(17) $$\begin{array}{l} \bar{UA}=\sqrt{{\bar{US}}^{2}-{\bar{SA}}^{2}}\\ △\varphi =\arctan\frac{\bar{SA}}{\bar{UA}}\\ \bar{SA}=r_{obs} \end{array}$$

where $\bar{UA}$, $\bar{US}$ and $\bar{SA}$ represent the length of line segment UA, US and SA, respectively.

If the heading angle $\psi_{i}$ of the UAV *i* in the range of [φ−Δφ,φ+Δφ], it is necessary to consider the impact of the obstacle. Therefore, the UAV *i* needs to take action to avoid this obstacle.

### 3.3. Mapping of the Intelligent Behavioral Patterns of Starlings

Suppose a system that consists of *n* UAVs in a three-dimensional Euclidean space. The dynamics of the UAVs are modeled as second-order integrators as follows:

(18) $$\left\{ \begin{array}{l} {\dot{q}}_{i}=p_{i}\\ {\dot{p}}_{i}=u_{i} \end{array}\right.,i=1,2,\ldots ,n$$

where $q_{i},\;p_{i}$ and $u_{i}\in R^{3}$ denote the position, velocity and control input vectors of the *i*-th UAV, respectively.

UAVs select their neighbors according to the principle called nearest-neighbor distance (as shown in Equation (1)). The desired geometric model of the flock requires that each UAV in the group keep the same distance from all its neighbors:

(19) $$\left\|q_{i}-q_{j}\right\|=d,\forall i,j\in N_{i}(t)$$

where *d* is a positive constant that represents the distance between UAV *i* and UAV *j*.

Based on the flocking algorithm [9] and behavioral pattern of starlings [36], the cooperative control law in a multi-obstacle environment is designed as follows:

(20) $$u_{i}=f_{i}^{nei}+f_{i}^{obs}+f_{i}^{neg}$$

where $f_{i}^{nei}$ is used to control UAVs to maintain the flocking geometry; $f_{i}^{obs}$ is adopted to control UAVs to avoid obstacles; $f_{i}^{neg}$ is applied to control UAVs to follow the navigation function.

Mapping the behavioral pattern mechanisms of starlings on a fixed-wing UAV. The formation of the UAV swarm is inspired by the collective patterns of starlings, which is described as follows:

(21) $$f_{i}^{nei}=c_{3}\underset{f_{i}^{pos}}{\underset{︸}{\sum_{j\in N_{i}(t)}(\frac{1}{\left\|q_{i}-q_{j}\right\|}-\frac{1}{{\left\|q_{i}-q_{j}\right\|}^{3}})}}-c_{4}[\beta \cdot \underset{f_{i}^{vel}}{\underset{︸}{\sum_{j\in N_{i}(t)}\left(p_{i}-p_{j}\right)}}+(1-\beta )\cdot \underset{f_{i}^{lof}}{\underset{︸}{\left(p_{i}-p_{l_{i}}\right)}}]$$

where $c_{3},c_{4}$ are positive coefficients; $p_{l_{i}}\in R^{3}$ is the velocity of the local leader in the neighbor set (refer to Equations (9)–(12) to select a local leader); β is the weight coefficient. If there is a local leader, individuals in the neighbor set will only follow the local leader and ignore the influence of other neighbors (β=0). Otherwise, the individual considers the movements of all the neighbors in the neighbor set (β=1).

The evasion pattern of the UAV is used to avoid an obstacle as follows:

(22) $$\begin{array}{l} f_{i}^{eva}\overset{\mathrm{mapping}}{\to }f_{i}^{obs}\\ f_{i}^{obs}=-\gamma \cdot c_{5}(\frac{q_{i}^{t-1}-q_{i}^{t}}{\left\|q_{i}^{t-1}-q_{i}^{t}\right\|}) \end{array}$$

where $c_{5}$ is a positive control gain. When the UAV *i* is avoiding the obstacle, γ=1. Otherwise, γ=0.

A virtual leader is set for the sake of making all UAVs move together. The navigational tracking force is introduced as follows:

(23) $$f_{i}^{neg}=-(1-\gamma )\cdot [c_{6}(q_{i}-q_{L})+c_{7}(p_{i}-p_{L})]$$

where $c_{6},c_{7}$ are positive coefficients; $q_{L},p_{L}\in R^{3}$ are the position and velocity of the virtual leader, respectively.

**Figure 8:**
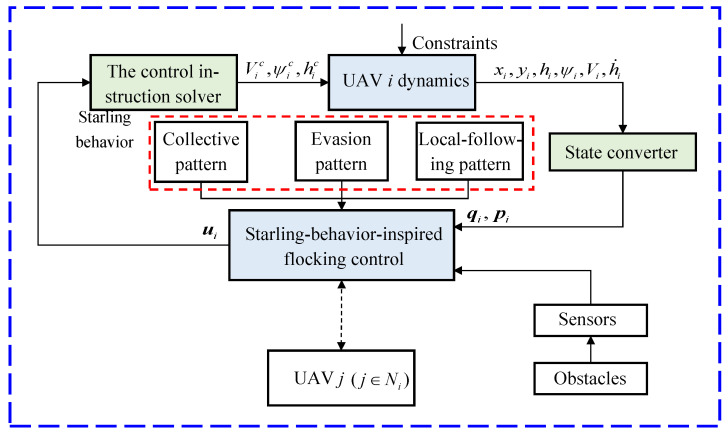
The block diagram of the control system for the UAV swarm.

As illustrated in Figure 8, the control framework for the obstacle avoidance of the UAV swarm is composed of two major parts, the fixed-wing UAV model and the proposed algorithm, and two auxiliary parts, the control instruction solver and the state converter.

The control instruction solver is used to transfer the control signal vector $u_{i}={\left[\begin{array}{ccc} u_{i}^{x} & u_{i}^{y} & u_{i}^{h} \end{array}\right]}^{T}$ to the control command of autopilots ${\left[\begin{array}{ccc} V_{i}^{c} & \psi_{i}^{c} & h_{i}^{c} \end{array}\right]}^{T}$. The specific solution equation is as follows:

(24) $$\left[\begin{array}{c} V_{i}^{c}\\ \psi_{i}^{c}\\ h_{i}^{c} \end{array}\right]=\left[\begin{array}{ccc} \tau_{v}\cos\psi_{i} & \tau_{v}\sin\psi_{i} & 0\\ -\frac{\tau_{\psi }}{V_{i}}\sin\psi_{i} & \frac{\tau_{\psi }}{V_{i}}\cos\psi_{i} & 0\\ 0 & 0 & \tau_{h} \end{array}\right]\left[\begin{array}{c} u_{i}^{x}\\ u_{i}^{y}\\ u_{i}^{h} \end{array}\right]+\left[\begin{array}{c} V_{i}\\ \psi_{i}\\ h_{i}+\frac{\tau_{h}}{\tau_{\dot{h}}}\dot{h} \end{array}\right]$$

The state converter is defined as follows:

(25) $$\left\{ \begin{array}{l} q_{i}={\left[\begin{array}{ccc} x_{i} & y_{i} & h_{i} \end{array}\right]}^{T}\\ p_{i}={\left[\begin{array}{ccc} V_{i}\cos\psi_{i} & V_{i}\sin\psi_{i} & {\dot{h}}_{i} \end{array}\right]}^{T} \end{array}\right.$$

The pseudo-code of the starling-behavior-inspired flocking control algorithm for UAVs is shown in Algorithm 1.


**Algorithm 1.** Starling-behavior-inspired flocking control algorithm for UAVs/*Initialization*/ Set initial parameters of the proposed algorithm and the model of fixed-wing UAV Generate the position $x_{i},y_{i},h_{i},\;\mathrm{heading}\;\mathrm{angle}\;\psi_{i}\;\mathrm{and}\;\mathrm{velocity}\;V_{i},\;{\dot{h}}_{i}$ of UAV *i* randomly /*Begin*/ **for** *i* = 1 to *n*  **for** *j* = 1 to *n*    Select neighbors according to Equation (1)    **if** UAV *j* is the neighbor of UAV *i*       UAV *i* interact with UAV *j* according to Equation (21)       
$\mathrm{Calculate}\;C_{ij}$ according to Equation (9)    **end if**    $\mathrm{Calculate}\;\varphi_{i}$ according to Equation (12)    $\mathrm{Find}\;\mathrm{the}\;\mathrm{local}\;\mathrm{leader}\;l_{i}$ according to Equations (10) and (11)  **end for**  $\mathrm{Calculate}\;R_{i}^{obs}$ to Equation (16)  $\mathrm{if}\;R_{d}\leq R_{i}^{obs}$   Execute collision prediction according to Equation (17)   $\mathrm{if}\;\varphi -\Delta \varphi \leq \theta_{i}\leq \varphi +\Delta \varphi$    Set parameter γ=1    Calculate the control signal of evasion pattern of UAV *i* according to Equation (22) **   end if**   **if** there exist a local leader *l_i_*    Set parameter *β* = 0    UAV *i* follows the local leader according to Equation (21)   **end if**  **end if**  Follow the virtual leader according to Equation (23)  /*Limitation*/  Set limitations according to Equation (15)  Update the position $q_{i}$ and velocity $p_{i}$ according to Equations (24) and (25) **end for**


### 3.4. Conversion of Patterns

In Section 2, behavioral patterns that emerge in a flock of starlings have been described carefully. However, when mapping behavioral patterns on a swarm of fixed-wing UAVs, the conversion of these patterns is essential to maintain the motion stability of the swarm of fixed-wing UAVs. In a state without external stimuli, the UAV swarm will be in the collective pattern. When a UAV has entered the scope of an obstacle (i.e., $R_{d}\leq R_{i}^{obs}$), the collision prediction mechanism is applied to predict whether the UAV will collide with the obstacle or not. If the collision prediction mechanism has determined that the UAV will hit the obstacle (i.e., $\varphi -\Delta \varphi \leq \psi_{i}\leq \varphi +\Delta \varphi$), this UAV will activate the evasion pattern to avoid the obstacle. In a neighbor unit, the UAV performing a sudden turning action (which indicates this UAV may have detected threats, i.e., $\max C_{ij}\geq C^{*}$) will be selected as a local leader to lead other UAVs in this unit to avoid potentially dangerous obstacles.

**Figure 9:**
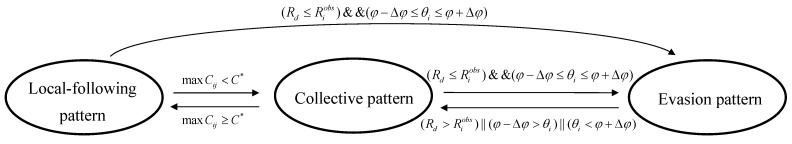
The conversion of patterns.

A brief figure about conversion of patterns of a single UAV *i* is depicted in Figure 9.

## 4. Simulation Results and Analysis

In this section, several simulation results of a fixed-wing UAV swarm in a 3D obstacle environment have been presented. For the performance assessment of the proposed algorithm, the basic flocking algorithm [9] (the method of obstacle avoidance is APF) and the proposed algorithm are tested in an obstacle-dense environment, a dynamic obstacle environment and an obstacle environment with both static and dynamic obstacles. Controlled objects used in all simulations are a UAV swarm consisting of *n* = 50 small fixed-wing UAVs. Each UAV has a range sensor with a limited measuring range *R* without having prior information about the experimental scenario. The UAV swarm tracks the trajectory of the virtual leader to reach the destination. The start point of the virtual leader is $q_{L}(0)={\left[\begin{array}{ccc} 0 & 0 & 0 \end{array}\right]}^{T}$. The initial states are set randomly as $x_{i}(0)\in [0,10]$, $y_{i}(0)\in [0,10]$, $V_{i}(0)\in [0,10]$, ${\dot{h}}_{i}(0)\in [0,1]$, $\psi_{i}(0)\in [-\pi /2,\pi /2]$.

Table 1 and Table 2 display values of parameters of the proposed algorithm and the model of fixed-wing UAV, respectively.

### 4.1. Performance Metrics

To evaluate the performance of the proposed algorithm during flight in an obstacle environment, several metrics [40,41] are defined as follows:

(1) Order parameter: it captures the coordination of the motion of the UAV swarm and represents the velocity alignment degree of all UAVs in the swarm.
  (26) $$\Phi =\frac{1}{n(n-1)}\sum_{i,j\neq 1}\frac{v_{i}\cdot v_{j}}{\left\|v_{i}\right\|\left\|v_{j}\right\|}$$
  where Φ varies from [0, 1]. Φ=1 indicates that the UAV swarm is in an ordered state while Φ=0 indicates that the UAV swarm is in a chaotic state.
(2) Safety metrics: it measures the risk of collision between the UAV swarm and obstacles and assesses the ability of the UAV swarm to avoid collisions with the obstacles.
  (27) $$\Phi_{s}=1-\frac{n_{obs}}{n}$$
  where the number of UAV that enters the obstacle zone is $n_{obs}=\left|\left\{\left(i,m\right)\wedge R_{i}^{obs}<r_{obs}\right\}\right|$. $\Phi_{s}=1$ reveals that no UAVs enter the obstacle zone while $\Phi_{s}=0$ reveals that the whole UAV swarm enters the obstacle zone.
(3) Tracking error: it evaluates the tracking performance of the UAV swarm. The tracking error of position is error between the position of UAV swarm and virtual leader, which can be described as follows:
  (28) $$\begin{array}{l} e_{q}=\left\|\langle q\rangle -q_{L}\right\|\\ \langle q\rangle =\frac{1}{N}\sum_{i=1}^{N}q_{i} \end{array}$$
  The tracking error of altitude is error between the altitude of UAV swarm and virtual leader, which can be described as follows:
  (29) $$\begin{array}{l} e_{h}=\left\|\langle h\rangle -h_{L}\right\|\\ \langle h\rangle =\frac{1}{N}\sum_{i=1}^{N}h_{i} \end{array}$$

### 4.2. Simulation in Obstacle-Dense Environment

**Figure 10:**
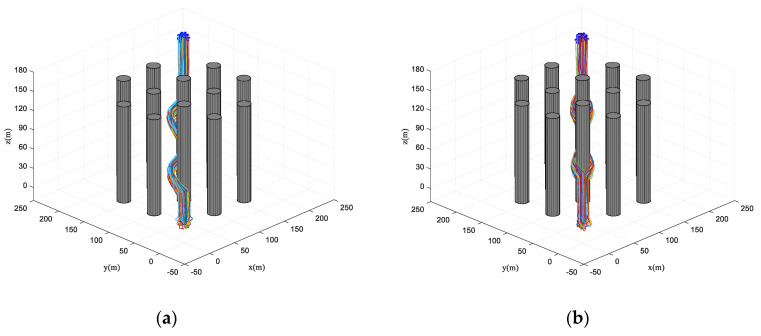

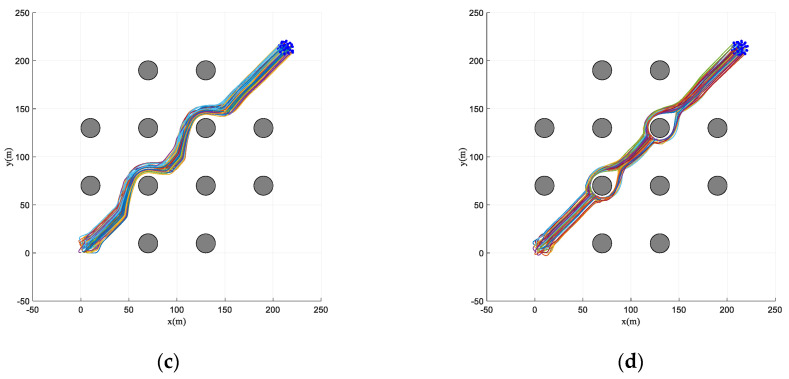
Flight paths in obstacle-dense environment. (**a**,**c**) are trajectories using the proposed algorithm in 3D space and *x*-*y* plane. (**b**,**d**) are trajectories using the basic flocking algorithm in 3D space and *x*-*y* plane.

First, the performance of the proposed algorithm is preliminarily tested in an obstacle-dense environment. The destination of the virtual leader is ${\left[\begin{array}{ccc} 220 & 220 & 160 \end{array}\right]}^{T}$ and the velocity of the virtual leader is $p_{L}(t)={\left[\begin{array}{ccc} 6 & 6 & 4 \end{array}\right]}^{T}$. The mission requires the fixed-wing UAV to track the trajectory of the virtual leader in an obstacle-dense environment with cylinder obstacles (the radius of each cylinder obstacle is 10 m and the number of obstacles *k* = 12). Flight paths in the 3D space and *x*-*y* plane are shown in Figure 10. In Figure 10a–d, it is obvious to see that each UAV is randomly distributed, i.e., the UAV swarm is in a disorder state at the beginning of the simulation. The UAV swarm stays in the collective pattern before entering the obstacle zone. Therefore, the UAV swarm reaches the consistent state immediately. In addition, it can be seen that the flight paths of the proposed algorithm have a smaller curvature compared with those of the basic flocking algorithm, which is beneficial to the stable operation of the UAV swarm.

**Figure 11:**
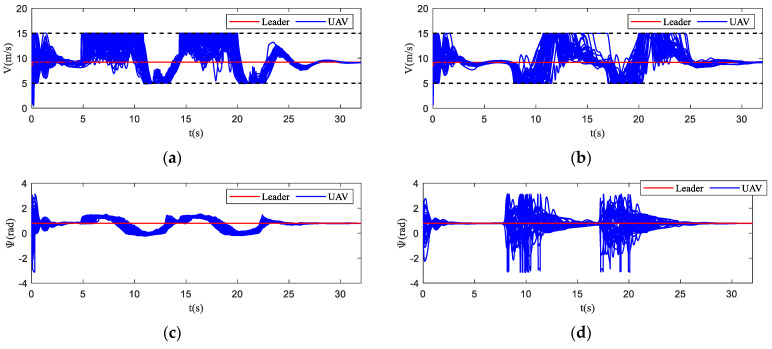

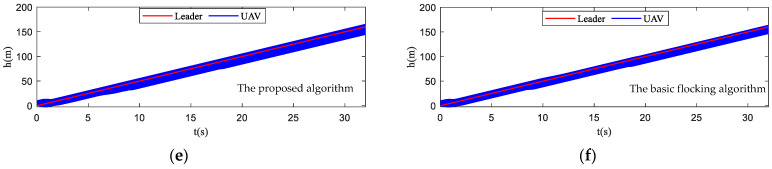
The time–response curves of velocity, heading angle, altitude for each UAV and virtual leader in obstacle-dense environment. (**a**,**c**,**e**) are the results using the proposed algorithm; (**b**,**d**,**f**) are the results using the basic flocking algorithm.

**Figure 12:**
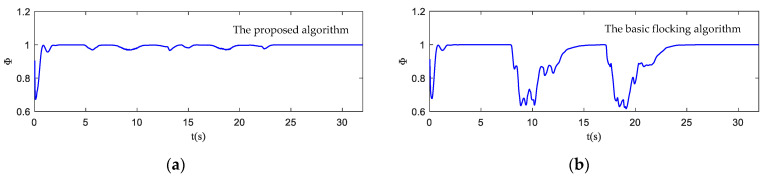
The order parameter curves of UAV swarm in obstacle-dense environment. (**a**) is the result using the proposed algorithm; (**b**) is the result using the basic flocking algorithm.

**Figure 13:**
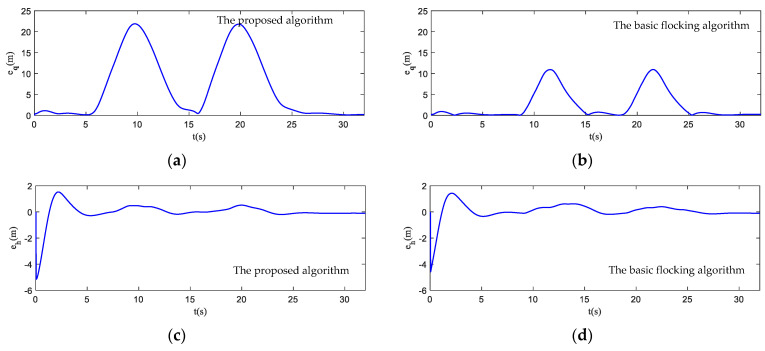
Tracking errors of position and altitude curves in obstacle-dense environment. (**a**,**c**) are tracking error curves using the proposed algorithm while (**b**,**d**) are tracking error curves using the basic flocking algorithm.

The motion state histories of each UAV and the virtual leader are shown in Figure 11, including the velocity, heading angle and altitude. As shown in Figure 11, the time–response curve of velocity, heading angle and altitude of each UAV will converge when the UAV swarm is close to the destination. When the UAV swarm enters the obstacle zone, the evasion pattern is started. As displayed in the simulation results, the oscillations of the heading angles of UAVs using the proposed algorithm are weaker than those using the basic flocking algorithm when avoiding obstacles, which indicates that the proposed algorithm can help to implement a smoother path for obstacle avoidance. Figure 12a,b show history of the order parameter of the proposed algorithm and the basic algorithm, respectively. It can be viewed that the fluctuation of the order parameter curve for the proposed algorithm is less drastic than that for the basic flocking algorithm, which further demonstrates that the proposed algorithm is better at maintaining formation when avoiding obstacles. The position tracking error curve (shown in Figure 13) of using the proposed algorithm is larger than using the basic flocking algorithm due to the fact that a larger distance from obstacles will be kept when using the proposed algorithm.

**Figure 14:**
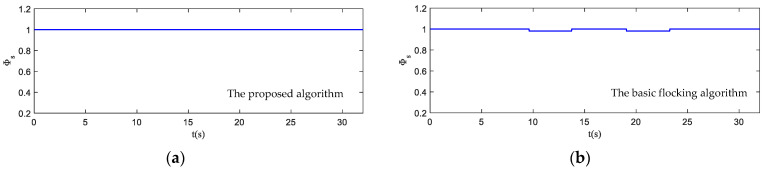
The safety parameter curves of the UAV swarm in obstacle-dense environment. (**a**) is the result using the proposed algorithm; (**b**) is the result using basic flocking algorithm.

**Figure 15:**
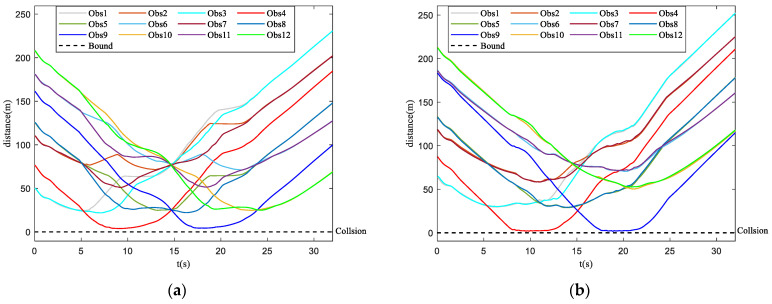
Curves of minimum distances between obstacles and each UAV in obstacle-dense environment. (**a**) is the result using the proposed algorithm; (**b**) is the result using the basic flocking algorithm.

Figure 14a,b are safety parameter curves during obstacle avoidance. Figure 15a,b display the minimum curve distance between obstacles from obstacle one (Obs1) to obstacle twelve (Obs12) and each UAV in the UAV swarm. The UAV swarm only avoids obstacles that have been detected as a collision risk by collision prediction. It can be seen that some UAVs have entered the obstacle zone while using the basic flocking algorithm (as shown in Figure 14b) and the minimum distance of Obs4, Obs9 in Figure 15b is closer to the collision-bound UAVs when compared with Figure 15a, which indicates that UAVs using the proposed algorithm are less likely to collide with obstacles.

### 4.3. Simulation in Dynamic Threat Environment

**Figure 16:**
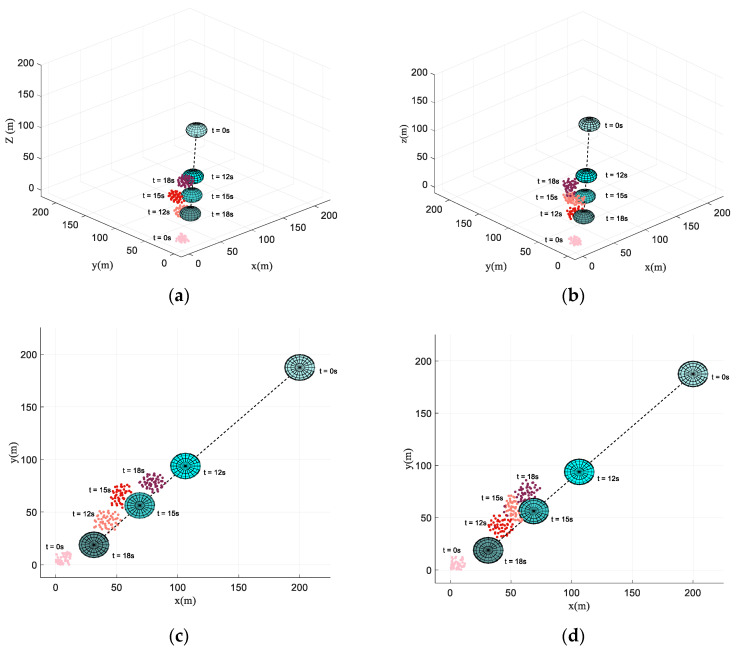
Snapshots of the motion of the UAV swarm and dynamic threat at t = 0 s, t = 12 s, t = 15 s, t = 18 s. (**a**,**c**) are the results using the proposed algorithm, while (**c**,**d**) are the results using the basic algorithm. (**a**,**b**) are in the 3D space while (**c**,**d**) are in the horizontal plane.

In this scenario, a dynamic threat with the velocity of 15 m/s is set. The mission of the UAV swarm and parameters are the same as those in the obstacle-dense environment. Figure 16 illustrates the formation producing and obstacle avoidance process using the proposed algorithm and the basic flocking algorithm, respectively. Each dot in Figure 16 represents a UAV and the black dotted lines represent the trajectory of the dynamic threat. The colors of the UAV swarm and dynamic threat vary from light to dark with the increase in simulation time. At beginning of the simulation, the UAVs’ positions and heading angles distribute randomly within the given range. In the collective pattern, the formation of the UAV swarm has formed.

During the process of obstacle avoidance, the consistency of the UAV swarm using the proposed algorithm is better than using the basic flocking algorithm. In Figure 16d, it is not hard to identify that several UAVs even disperse from the UAV swarm.

**Figure 17:**
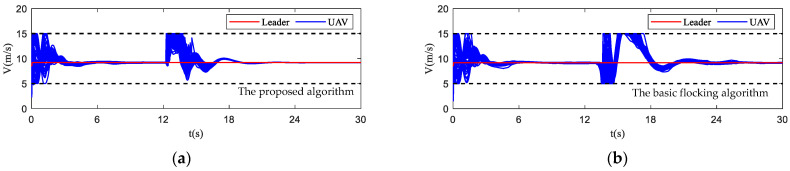

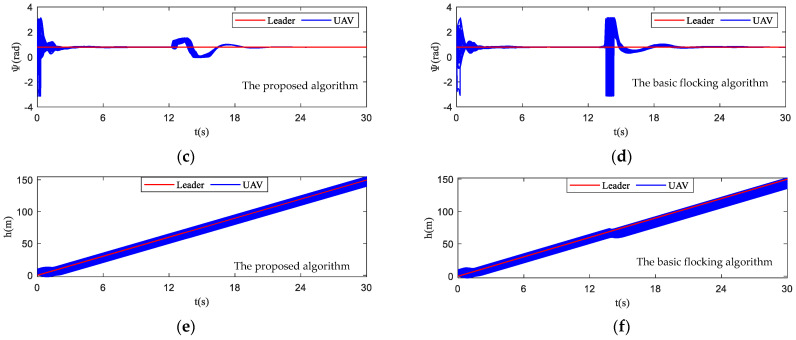
The time–response curves of velocity, heading angle, altitude for each UAV and virtual leader in dynamic threat environment. (**a**,**c**,**e**) are the results using the proposed algorithm; (**b**,**d**,**f**) are the results using the basic flocking algorithm.

**Figure 18:**
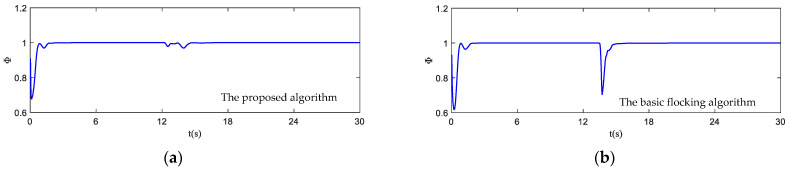
The order parameter curves of UAV swarm in dynamic threat environment. (**a**) is the result using the proposed algorithm; (**b**) is the result of using the basic flocking algorithm.

**Figure 19:**
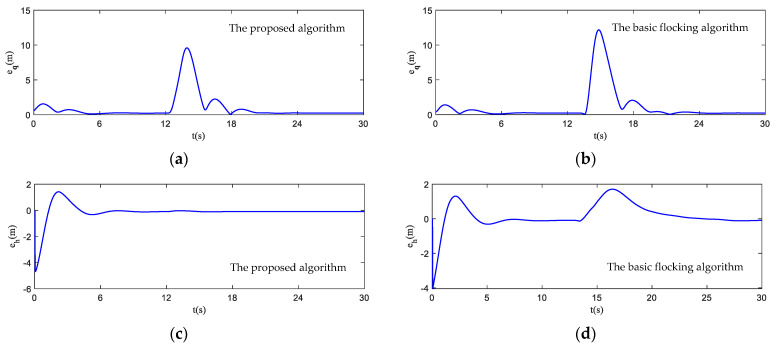
Tracking errors of position and altitude curves in dynamic threat environment. (**a**,**c**) are tracking error curves using the proposed algorithm, while (**b**,**d**) are tracking error curves using the basic flocking algorithm.

In Figure 17, the motion histories of each UAV and the virtual leader, including the velocity, heading angle and altitude, are displayed. It can be seen that after the process of obstacle avoidance, the curves of velocity and heading angle converge faster in the proposed algorithm. However, this advantage is not obvious in the static obstacle environment (Figure 11). Therefore, the proposed algorithm has more advantages in a dynamic threat environment. The oscillation of heading angles of the basic flocking algorithm is still stronger than that of the proposed algorithm, which is further confirmed by the order parameter stated in Figure 18. Tracking error curves shown in Figure 19 illustrate that both the tracking errors of position and altitude of the proposed algorithm are less than that of the basic flocking algorithm. This phenomenon indicates that the proposed algorithm is has a better tracking performance when it is applied to the dynamic threat environment than when it is applied to the static threat environment (Figure 13).

**Figure 20:**
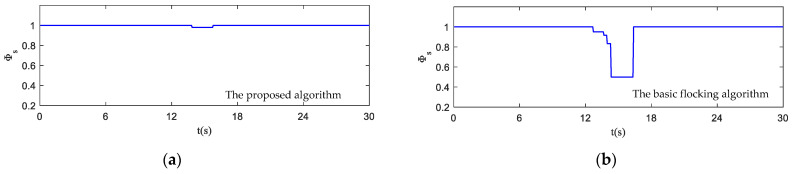
The safety parameter curves of the UAV swarm in dynamic threat environment. (**a**) is the result using the proposed algorithm; (**b**) is the result using basic flocking algorithm.

**Figure 21:**
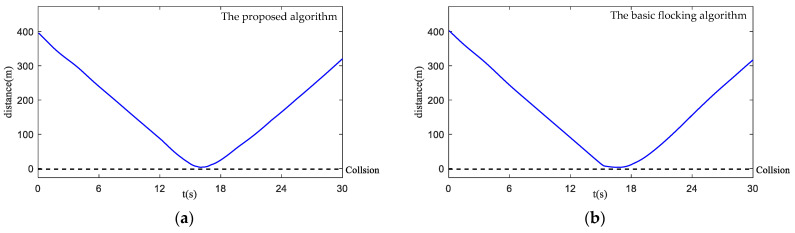
Curves of minimum distances between dynamic threat and each UAV in the UAV swarm. (**a**) is the result using the proposed algorithm; (**b**) is the result using basic flocking algorithm.

Figure 20a,b show the safety parameter curves of the UAV swarm in the dynamic threat environment. Curves of minimum distance between the dynamic threat and each UAV in the UAV swarm are shown in Figure 21a,b. It is noticeable that more UAVs in the UAV swarm enter the obstacle zone when using the basic flocking algorithm. Therefore, the security of the UAV swarm is guaranteed when using the proposed algorithm.

### 4.4. Simulation in Obstacle Environment with Static and Dynamic Obstacles

**Figure 22:**
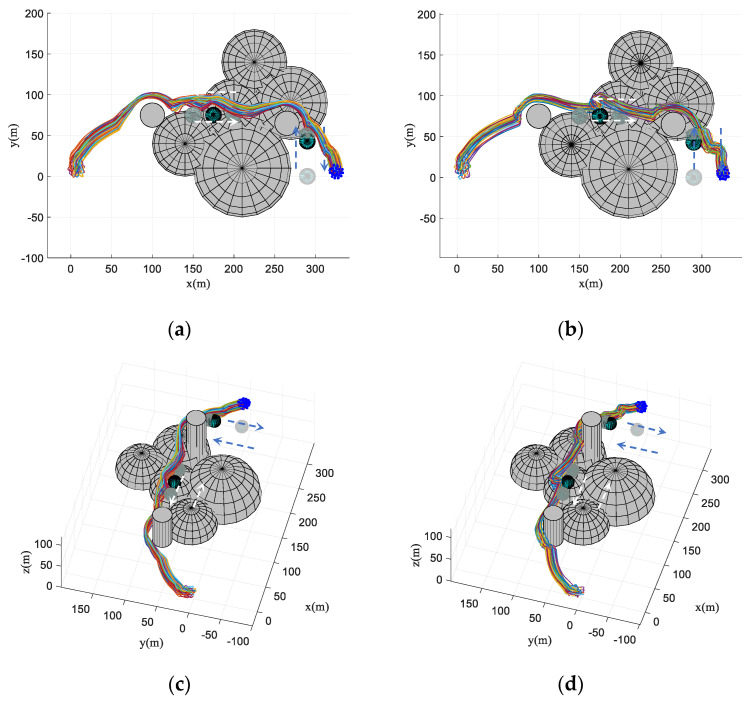
Flight paths in obstacle environment with static and dynamic obstacles. (**a**,**c**) are trajectories using the proposed algorithm in 3D space and *x*-*y* plane. (**b**,**d**) are trajectories using the basic flocking algorithm in 3D space and *x*-*y* plane.

**Figure 23:**
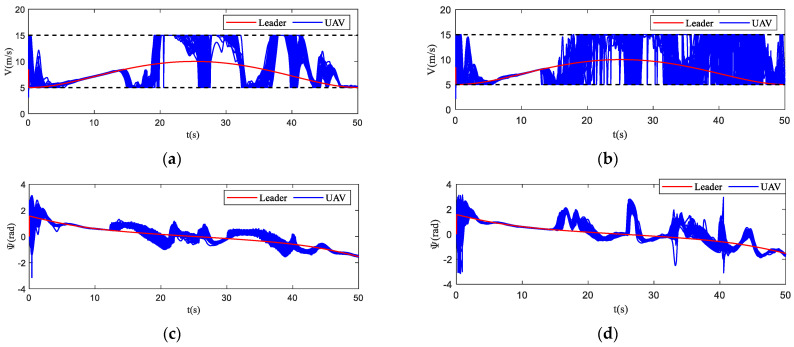

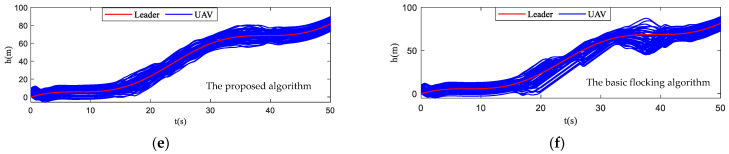
The time–response curves of velocity, heading angle, altitude for each UAV and virtual leader in obstacle environment with static and dynamic obstacles. (**a**,**c**,**e**) are the results using the proposed algorithm; (**b,d**,**f**) are the results using the basic flocking algorithm.

**Figure 24:**
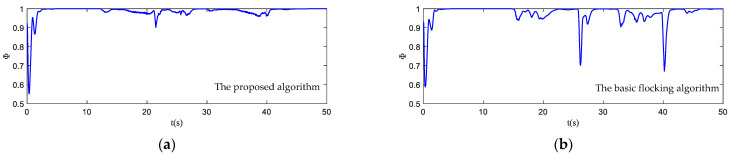
The order parameter curves of UAV swarm in obstacle environment with static and dynamic obstacles. (**a**) is the result using the proposed algorithm; (**b**) is the result using the basic flocking algorithm.

**Figure 25:**
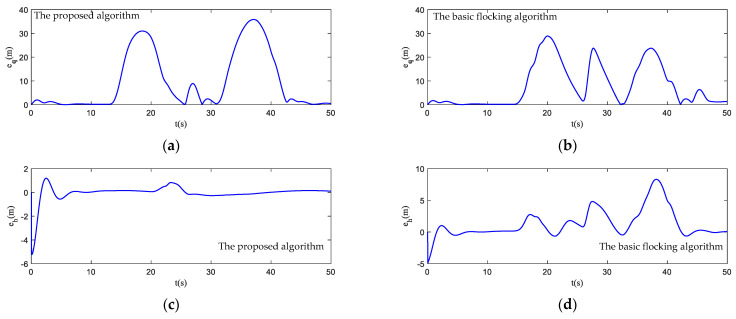
Tracking errors of position and altitude curves in obstacle environment with static and dynamic obstacles. (**a**,**c**) are tracking error curves using the proposed algorithm, while (**b**,**d**) are tracking error curves using the basic flocking algorithm.

**Figure 26:**
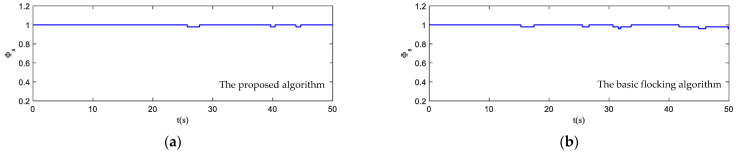
The safety parameter curves of the UAV swarm in obstacle environment with static and dynamic obstacles. (**a**) is the result using the proposed algorithm; (**b**) is the result using basic flocking algorithm.

**Figure 27:**
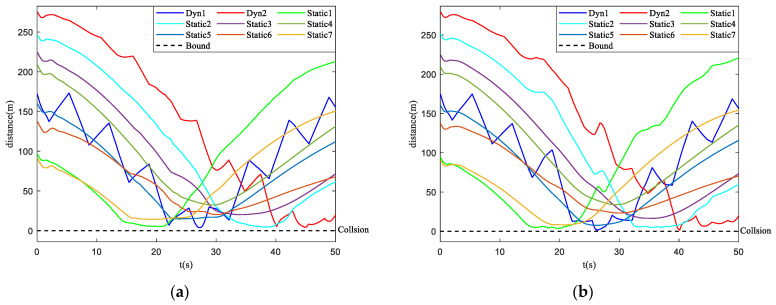
Curves of minimum distances between obstacles and each UAV in obstacle environment with static and dynamic obstacles. (**a**) is the result using the proposed algorithm; (**b**) is the result using the basic flocking algorithm.

In this simulation, the trajectory of the virtual leader is an elliptical shape and the velocity of the virtual leader is $p_{L}(t)={\left[\begin{array}{ccc} 10\sin(t/16) & 5\cos(t/16) & -2\sin(t/5) \end{array}\right]}^{T}$. The parameters are the same as those in the dynamic threat environment simulation. The scenario for this experiment is composed of seven static obstacles (Static 1–Static 7) and two dynamic obstacles. Dynamic obstacle one (Dyn1) moves back and forth between point [150, 75, 50] and point [200, 75, 50] along the *x*-axis, while dynamic obstacle two (Dyn2) moves back and forth between point [295, 0, 70] and point [295, 50, 70] along the *y*-axis. The basic flocking algorithm and the proposed algorithm are both implemented for comparison. The flight paths of each UAV in both the horizontal plane and the 3D plane are displayed in Figure 22, which reflects that the flight paths of UAVs using the proposed algorithm are smoother than those of UAVs using the basic flocking algorithm when the UAV swarm encounters dynamic obstacles. The corresponding state response curves and history of order parameter are shown in Figure 23 and Figure 24, respectively. Tracking errors of position and altitude curves are displayed in Figure 25. From the simulation results, UAVs using both the proposed algorithm and the basic algorithm can track the desired trajectory. However, UAVs using the proposed algorithm are able to accomplish this mission with a more ordered formation and less fluctuation of velocity and altitude. The history of safety parameters and curves of minimum distances between obstacles and each UAV in the UAV swarm are shown in Figure 26 and Figure 27, respectively. By virtue of the local-following pattern, the UAV swarm is able to maintain a safer distance from obstacles when using the proposed algorithm.

## 5. Conclusions

This paper investigates the problem of obstacle avoidance of a UAV swarm in an unknown 3D environment by mimicking the behavior patterns of starlings. A flock of starlings exhibits high consistency and flexibility when evading threats. Behavioral mechanisms which emerged in flocks of starlings including collective pattern, evasion pattern and local-following pattern are mapped on the UAV swarm to improve the performance of obstacle avoidance. The collective pattern utilizes neighbor selection and interaction to guarantee the geometric shape of the UAV swarm; the evasion pattern is applied to avoid collision with obstacles in unknown environments and the local-following pattern selects a local leader to increase the efficiency of information transmission and enhance safety when the UAV swarm confronts dynamic threats. The practical dynamic constraints of a fixed-wing UAV, such as velocity limitation, etc., are taken into consideration in this paper. The proposed algorithm is comprehensively evaluated in three simulation scenarios, including an obstacle-dense environment, a dynamic threat environment and an unknown environment with both static and dynamic obstacles. Several metrics are set to the quantify the performance of both the proposed algorithm and basic flocking algorithm. The simulation results confirm that the proposed algorithm performs better on formation maintenance, velocity alignment when avoiding obstacles and the maintenance of safe distances from obstacles compared with the basic flocking algorithm. In the future, our work will focus on evaluating the effectiveness of the proposed algorithm on other unmanned systems such as unmanned ground vehicles (UGVs), autonomous underwater vehicles (AUVs) and so on. In addition, the proposed algorithm will be applicated on a physical platform to solve the actual obstacle avoidance problem.

## Figures and Tables

**Table 1:** Parameters in the proposed algorithm.

Parameter	Value
Sensing range, *R*	15 m
Desired distance between neighboring UAVs, *d*	5 m
Desired distance between UAV and obstacle, $R_{d}$	15 m
Coefficient for the selection of local leader, α	2.5
Control gains of local-following pattern, $c_{1},c_{2}$	0.5, 2
Step-size, Δt	0.025 s
Control gains of collective pattern, $c_{3},c_{4}$	1, 4
Control gains of evasion pattern, $c_{5}$	1
Control gains of virtual leader-follower, $c_{6},c_{7}$	1, 2

**Table 2:** Parameters of the model of the fixed-wing UAV.

Parameter	Value
Velocity time constant, $\tau_{v}$	5
Heading angle time constant, $\tau_{\psi }$	0.75
Altitude time constant, $\tau_{\dot{h}},\tau_{h}$	0.3, 1
Minimum and maximum velocity, $v_{\min},v_{\max}$	5 m/s, 15 m/s
Maximum lateral overload, $n_{\max}$	5 g
Maximum climbing and gliding velocity, $\lambda_{\mathrm{climb}},\lambda_{\mathrm{glide}}$	−5 m/s, 5 m/s

## Data Availability

Not applicable.
